# Drought risk probabilistic models based on extreme value theory

**DOI:** 10.1007/s11356-023-29093-5

**Published:** 2023-08-10

**Authors:** Arkadiusz Piwowar, Łukasz Kuźmiński

**Affiliations:** 1 Department of Economics and Organization of Food Economy, Wroclaw University of Economics and Business, Komandorska Street 118/120, 53-345 Wrocław, Poland; 2 Department of Process Management, Wroclaw University of Economics and Business, Komandorska Street 118/120, 53-345 Wrocław, Poland

**Keywords:** Climate extremes, Risk of drought, Probabilistic quantification, Environmental modelling, Extreme value theory

## Abstract

The article presents original, probabilistic models for the quantification of drought risk, based on generalized distribution functions for the distribution of maximum values as well as the standardized precipitation index. Using the models, a probabilistic measurement of drought risk has been made for three regions over four 5-year periods for each region. Three measurement points located in Poland were selected as a case study. The study provides an innovative approach in the field of probabilistic models of quantification; moreover, drought risk levels have been calculated for the selected locations. Furthermore, the method may be used for creating indexes for any climatic region subject to study.

## Introduction

Drought is an extreme climatic occurrence, a complex process which takes place in the water and soil environment. It yields a number of adverse effects on the functioning of the environment and the economy (Felbermayr et al. 2022; Oh et al. 2023; Xu et al. 2023). In the subject literature, drought is characterized by a multitude of definitions. Several different types are distinguished, the most common being meteorological drought, soil or agricultural drought, hydrological drought and socio-economic drought (Soule 1990; Keyantash and Dracup 2002; Mishra and Singh 2010; Zargar et al. 2011; Maity et al. 2016; Wu et al. 2018). In terms of the natural environment, drought leads to a decrease in water reserves in an ecosystem and water deficiency in catchment areas. This in turn creates an immediate socio-economic threat, with agriculture being the most exposed area (Lin et al. 2013; Geng et al. 2015; Ziolkowska 2016; Tigkas et al. 2019; Yang et al. 2023). Drought affects the functioning of agricultural ecosystems and the physiology of changes in plants, and the interactions between plants and soil microbe colonies are altered (Gupta et al. 2020; Williams and Vries 2020). A negative water balance in the soil is also an environmental threat for trees and forest stands. According to forecasts, drought-related tree mortality will increase along with climate change (Powers et al. 2020). However, difficulties in addressing the demand for water are not only limited to agriculture and forestry, but also affect, among others, the economy and industry.

Extreme weather phenomena, including disastrous drought and rainfall, are becoming ever more common and intense. Climate change is occurring faster than that anticipated by a range of forecasts, with hydrological phenomena often being extreme. An increase in the frequency of the occurrence of extreme phenomena is becoming a significant problem in many regions of the world (including Australia, South America and Europe) (Williges et al. 2017; Brito et al. 2018; King et al. 2020; Findlay 2020; Garreaud et al. 2020). In 2018, Central Europe witnessed the longest summer periods of drought and heat waves ever recorded (Schuldt et al. 2020; Boergens et al. 2020). Moreover, it is predicted that the frequency of drought will increase by season across the entire European continent (Spinoni et al. 2018). Currently, it has become necessary to expand drought monitoring programs in countries such as Poland (this paper includes measurements taken in three different locations in Poland).

Due to its fundamental significance for environmental, economic and social spheres, drought-related issues are becoming increasingly popular with interdisciplinary research teams. The usefulness of several different statistical methods and techniques is being looked into as part of attempts to determine the changing nature of hydrological conditions. This is very important in terms of the action that needs to be taken to prepare a region for drought and/or to mitigate the effects of drought (Guo et al. 2020; Dai et al. 2020). Three measurement points located in Poland were selected as a case study (these are described later).

The main aim of the research is to assess the risk of damage caused by drought in selected regions using an original probabilistic model of drought risk. This work describes the use of extreme value theory with regard to drought risk measurement.

Considering the aforementioned assumptions, the authors of the study have developed the two following research questions:

- How can the risk of extreme drought be measured?
- How can the threat of extreme drought effects be compared across different geographical locations?

In order to find answers to the research questions and to achieve the main goal of the research, four research hypotheses are proposed:

- *H1*: The distribution function of the generalized distribution of extreme values is very efficient in describing the probability of the occurrence of extreme drought, measured using the standardized precipitation index.
- *H2*: Local extreme drought phenomena correspond to mesoscale trends.
- *H3*: The risk level of extreme drought-related damage is characterized by significant variability in terms of time and space.
- *H4*: The probabilistic model of drought risk, based on the distribution function of the distribution of the standardized precipitation index, is universal by nature and may be used for measuring drought in regions located in different climatic zones.

This study addresses the risk connected with the occurrence of a natural phenomenon with possible disastrous effects. Risk generated by natural phenomena in the environment is considered elementary within an actuary in a broad sense. If risk generated by the natural environment is common, it is referred to as disaster risk, which causes significant material and health damage, and is especially dangerous for the stability of the economy.

Disaster risk is described as a natural phenomenon or human activity that can entail a great number of separate risks and cause damage to health and property which, when accumulated, may reach a dangerous scale (Gepert 1971). The definition of disaster risk notes that it is characterized by the time of occurrence, range and scale of damage being difficult or even impossible to predict. The risk of drought and the damage caused by it have two components: deterministic and probabilistic. For the purposes of measuring the risk of drought using probabilistic models, the authors of the study have proposed a definition of drought risk (definition 1), as presented below.

The study is organized in the following way. After the “Introduction” and “Literature review” sections, the research methodology is presented, including original models of probabilistic quantification of drought risk that use a theoretical distribution function of the distribution of the random variable describing the phenomenon as the basis of the models. Next, using theoretical tools, analysis was conducted of measurements of drought risk based on actual data of daily precipitation. Finally, the conclusions are presented along with possibilities for using the presented calculations.

## Literature review

As mentioned in the “Introduction” section, extreme weather phenomena, including droughts, are having an increasing effect on the economy, causing a number of socio-economic consequences both on a global, regional and local scale. As a result, a great deal of research has been undertaken in this area in the field of risk analysis and assessment at both a theoretical (methodological) and practical (operational) level.

The approach to drought risk assessment is gradually evolving along with scientific progress, from a single-factor assessment to a multi-factor risk assessment. There have been developments in the area of methodology, and expansion of the perspective for describing phenomena in terms of time and space, taking into account many factors as well as the intensity of the course. In simple analyses regarding the problem of drought, the standardized precipitation index (SPI) (McKee et al. 1993) is applied, which is based solely on precipitation, and measures how much precipitation during a given period deviated from historically established norms (Zarch et al. 2015). Other indicators used for this subject include, for example, the standardized precipitation-evapotranspiration index (SPEI) (Vicente-Serrano et al. 2010) and the reconnaissance drought index (RDI) (Tsakiris et al. 2007). The SPEI, developed in 2010, is being used in a growing number of climatological and hydrological studies (Beguería et al. 2014). Apart from precipitation, the second indicator (RDI) takes into account an additional meteorological parameter—potential evapotranspiration (Tsakiris et al. 2007). There are also many examples in the literature of the basic indicators undergoing modification. For example, in order to improve the assessment of agricultural drought, Tigkas et al. (2017) proposed the effective reconnaissance drought index (eRDI). The main feature of this modification is the replacement of total precipitation with effective precipitation.

On the basis of the above-mentioned indicators, including the hybrid approach, appropriate comparisons are made between the accuracy of the selection of drought indicators for specific applications and the drought risk assessment (Blauhut et al. 2016). A lot of studies demonstrate the legitimacy and popularity of using the SPI to characterize droughts, as well as the possibility of using the stochastic properties of SPI time series to predict changes in drought classes (Costa 2011). This is important, for example, in the context of developing forecasting tools (Paulo and Pereira 2006). In this context, the theory of extreme values may be useful as a tool for statistical analysis of extreme events in risk management. This theory provides good support for theoretical and methodological applications, especially in the face of a wide range of uncertainties about the risk, variability and severity of drought (Xu et al. 2011).

Methods based on the extreme value theory are used with increasing frequency for modelling and predicting extreme phenomena (Arns et al. 2015; Charon 2015; Gomes and Guillou 2015). The vast subject literature illustrates the great amount of interest in the subject area and the wide range of possible uses. The use of extreme value theory and the fitting of extreme distributions are becoming increasingly common in climate research. Moreover, an ever-greater number of steps are being taken towards probabilistic assessment of changes in the thematic area of drought (Burke et al. 2010).

This study refers to a current of research related to probabilistic methods for modelling drought risk (Mishra et al. 2009; Hao et al. 2017; Ayantobo et al. 2018; Langat et al. 2019). Although significant progress has been made, the critical research problem posed in this paper has still not been the subject of advanced research (see hypotheses 1–4). It is particularly essential to be able to use the author’s solutions (presented later in the paper) for more effective risk assessment and, consequently, optimization in the area of drought management. The applied stochastic method, which uses elements of the theory of extreme values, requires a sufficiently large sample size for the estimation of model parameters in order to ensure the high quality of the obtained estimators. The drought risk model has been developed on the basis of one of the commonly used indexes for drought assessment—SPI—along with selected elements of extreme value theory. The presented literature review is the background for the subsequent considerations, especially in the next section. This presents detailed references to the literature on the subject in the context of developing a proprietary model of probabilistic quantification of the risk of damage caused by extreme drought.

## Materials and methods

The subject literature presents different methods for determining the SPI (McKee et al. 1993)*.* In practice, one method in particular is often used. The method uses the fact that if the random variable *X* has a Gamma distribution, its transform $Y=\sqrt[3]{X}$ has an approximately normal distribution (Krishnamoorthy et al. 2008). Using the aforementioned characteristic, the SPI for $x\ge 0$ may be determined with the following formula:

1 $${\text{SPI}}=\frac{Y-\widehat{\mu }}{\widehat{\sigma }}$$

where *Y* is the random variable whose values are monthly precipitation amounts after the transformation ($Y=\sqrt[3]{X}$) has been used, while $\widehat{\mu }$ and $\widehat{\sigma }$ are the average and the standard deviation for monthly precipitation amounts in the studied period (e.g. 5 years, as assumed in this study).

The classification of drought levels based on SPI, including precipitation conditions for the climatic location of Poland, was developed by Łabędzki (2006), and is presented in Table 1.

**Table 1:**
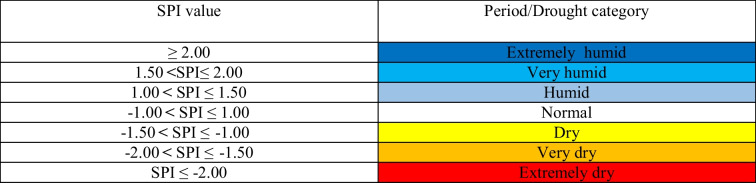
Classification of drought levels, based on SPI values

Using the stochastic approach in the proposed models for measuring drought-related damage, the SPI described with formula (1) is a random variable. For simplicity, the following designation of the index as a random variable has been used in the study: *Z* = SPI. According to the literature, the SPI has an approximately normal distribution.

In the study, the main random variable used for measuring drought risk is the variable *Z* = SPI. Having conducted preliminary research on the distribution of SPI in selected locations, the study authors made the decision to include the generalized extreme value distribution in the proposed models for drought risk.

The generalized extreme value distribution (GEV) is mainly used for modelling the distribution of random variables ${M}_{n}=\mathrm{max}\left\{{X}_{1},...,{X}_{n}\right\}$ which constitute maxima from a sequence* n* of independent random variables ${X}_{1},...,{X}_{n}$ with identical distributions. In the proposed models for drought risk, the theoretical distribution function of the distribution of maximum values will describe the distribution of random variable *Z* = SPI, which is the basis for probabilistic quantification of risk of drought-related damage.

The distribution function for the generalized extreme value distribution ${G}_{\gamma }$ proposed by Von Misess (1936) is given by the formula:

2 $$G_\gamma(x)=\left\{\begin{array}{ll}\exp\left\{-\left[1+\gamma x\right]^{-\frac1\gamma}\right\}&\mathrm{if}\;\;\gamma\neq0,\;1+\gamma x>0,\\\exp\;\left\{-\exp\left(x\right)\right\}&\mathrm{if}\;\;\mathrm\gamma=0,\;\mathrm x\in\mathrm R.\end{array}\right.$$

Distribution function ${G}_{\gamma }$ uses one formula to describe a family of three distribution functions for maximum borderline values determined in a fundamental theorem of extreme value theory, the Fisher and Tippett (1928) theorem.

Often, for ease of use, a version of the distribution function of the generalized extreme value distribution ${G}_{\gamma }$ is used, extended to include a location parameter *μ* and scale parameter *σ*. The parametrization of distribution function ${G}_{\gamma }$ is made on the basis of theorem 1 presented below.

**Theorem 1.** If random variable *X* has distribution function *F*, then random variable (*μ* + *σX*) has distribution function ${F}_{\mu ,\sigma }(x)=F((x-\mu )/\sigma )$ (Thomas and Reiss 2007).

According to theorem 1, after parametrization, distribution function ${G}_{\gamma }(x)$ is transformed into distribution function ${G}_{\gamma ,\mu ,\sigma }\left(\frac{x-\mu }{\sigma }\right)$, described with the following formula (Engeland et al. 2005; Kotz and Nadarajah 2005):

3 $$G_{\gamma,\mu,\sigma}(x)=\left\{\begin{array}{ll}\exp\left\{-\left[1+\gamma\left(\frac{x-\mu}\sigma\right)\right]^{-\frac1\gamma}\right\}&\mathrm{if}\;\;\gamma\neq0,\;1+\gamma\frac{x-\mu}\sigma>0\\\exp\left\{-\exp\left(\frac{x-\mu}\sigma\right)\right\}&if\;\gamma=0,\;x\in\mathrm R\end{array}\right.$$

The parameters of distribution function ${G}_{\gamma ,\mu ,\sigma }(x)$, that is $\gamma ,\mu {\;and}\;\sigma$, are particularly important for detailed analysis of the modelled phenomenon, and for comparison of phenomena over time and space.

Among the three parameters of distribution function ${G}_{\gamma ,\mu ,\sigma }(x)$, parameter *γ* is particularly important for studying drought risk. From a mathematical perspective, the parameter is called the shape parameter. In extreme value theory, this parameter is called the extreme value index (EVI). Statistically, its values describe the properties of the distribution tails of the modelled variable (for the sake of this study, the variable is *Z* = SPI). Values above zero yield thick distribution tails for the reference variables, and therefore, the probability of extreme phenomena is relatively high. A zero value yields a thin tail, while values below zero yield cut distribution in which some extreme values are not possible.

The subject literature suggests several methods of estimating the parameters of GEV distribution. The maximum likelihood method, along with the probability-weighted moment method, is one of the best and most often used methods of estimating the parameters of GEV distribution (Hosking et al. 1985). This study uses the linear combinations of ratios of spacing (LRSE) method for estimating the parameters of distribution function ${G}_{\gamma ,\mu ,\sigma }(x)$ (Johnson and Kotz 1970).

With research done in the last 20 years in particular, where random variables describing natural phenomena such as drought are used, it is necessary to take into consideration the dynamic climate change taking place around the world (Herman et al. 2020). Drought study practice analyses time series over long periods of time. Climate variability over long time horizons and with regular fluctuations significantly affects the parameters of the distribution function for the distribution of random variable *Z* = SPI describing the total monthly precipitation with reference to the average total monthly precipitation and its variability across the studied time horizon. That is why it is necessary to set time horizons for which the estimations will be done.

In this study, for each studied location, the authors have accepted a period of 5 years as a basic time horizon. This means that the parameters of the distribution function for the distribution of random variable Z, indicating the SPI for the studied location, have been estimated for the 5-year periods specified in the research. For each month of the studied 5-year period, the SPI was calculated with formula (1) using data that shows the total daily amounts of precipitation in mm. The SPI was calculated according to a procedure composed of the following steps:

1. Initially, based on data that shows total daily precipitation for all the 5-year periods studied and three locations, total monthly precipitation was calculated, expressed in random variable *X*.
2. Next, the expressions of random variable *X* were transformed into values of random variable $Y=\sqrt[3]{X}$.
3. Subsequently, for data that expresses random variable *Y*, i.e. transformed total monthly precipitation for every 5-year period in each of the three locations, the average ($\widehat{\mu }$) and standard deviation ($\widehat{\sigma }$) were calculated;
4. Using the values of random variable *Y*, the $\widehat{\mu }$ and $\widehat{\sigma }$ were calculated for each of the studied 5-year periods, along with the monthly SPI values (expression of random variable *Z*).

The data obtained was used to estimate the parameters of 12 distribution functions ${G}_{\gamma ,\mu ,\sigma }$ for distributions of random variables *Z* = SPI depicting the distributions of standardized precipitation indexes in four 5-year periods for each of the three analysed locations. The estimation resulted in the estimator vector $\left(\widehat{\gamma },\widehat{\mu },\widehat{\sigma }\right)$ being obtained for each of the twelve distribution functions. In other words, the time series of the expression of random variable *Z* was matched to the appropriate distribution function ${G}_{\gamma ,\mu ,\sigma }$, which resulted in obtaining the distribution parameters $\left(\widehat{\gamma },\widehat{\mu },\widehat{\sigma }\right)$ (Matthys and Beirlant 2001).

The obtained estimated distribution functions of the distributions of random variables *Z* = SPI form the basis for developing the probabilistic models proposed by the authors for measuring drought risk and drought-related damage. The models presented in what follows are an instrument for assessing the probability of total monthly precipitation occurring, measured with an SPI below a certain critical level* u*, described with the formula:

4 $$P\left(Z<{u}_{p}\right)=p, \mathrm{where}\;p\in \left(0,1\right)$$

where *u*_*p*_ is formally known as the *p-*quantile of the generalized GEV distribution. The value of the quantile is obviously the function of parameters of the distribution function of the distribution of maximum values ${G}_{\gamma ,\mu ,\sigma }$ (McNeil 1998). Ultimately, it depends on the value of the theoretical distribution function:

5 $$\begin{array}{c}{u}_{p}={G}_{\gamma ,\mu ,\sigma }^{-1}\left(p\right)=\widehat{\mu }-\frac{\widehat{\sigma }}{\widehat{\gamma }}\left(1-{\left(-\mathrm{ln}\left(p\right)\right)}^{-\widehat{\gamma }}\right),{\text{f}}{\text{o}}{\text{r}} \, \gamma \ne 0,\\ {u}_{p}={G}_{\gamma ,\mu ,\sigma }^{-1}\left(p\right)=\widehat{\mu }-\widehat{\sigma }\left(-\mathrm{ln}\left(p\right)\right),{\text{f}}{\text{o}}{\text{r}} \, \gamma =0.\end{array}$$

At this point in the study, it is necessary to present the definitions of drought risk and risk of drought-related damage for the sake of probabilistic quantification. The authors propose their own definition of drought risk for use in the proposed original measurement method using a model for probabilistic quantification of drought risk (definition 1).

**Definition 1**. Drought risk is potential damage resulting from random variable *Z* = SPI reaching a value below critical level *d*_cr_. Due to the fact that the random component of drought risk is subject to a certain distribution of probability, it is also possible to assess the probability of certain random phenomena. In order to measure drought risk, the authors propose a probabilistic measure of drought risk level (definition 2).

**Definition 2**. The probabilistic measure of drought risk level $D\left(G,{d}_{cr}\right)$ is defined as the probability of random variable *Z* exceeding critical level *d*_cr_,

6 $$D\left(G,{d}_{cr}\right)=P\left(Z<{d}_{cr}\right)={p}_{cr}$$

where *G* is the distribution function of the distribution of random variable *Z*. The distribution function* G* may be any theoretical distribution function that describes the distribution of random variable* Z* well enough. In the research presented in the study, the authors have accepted the distribution function of the generalized extreme value distribution ${G}_{\gamma ,\mu ,\sigma }$ or the distribution function of normal distribution, as the literature suggests, to be distribution function* G.*

Measure $D\left({G}_{\gamma ,\mu ,\sigma },{d}_{cr}\right)$, in which distribution function ${G}_{\gamma ,\mu ,\sigma }(z)$ was substituted, may be described with the formula:

7 $$D\left({G}_{\gamma ,\mu ,\sigma },{d}_{cr}\right)={G}_{\gamma ,\mu ,\sigma }\left({d}_{cr}\right)={p}_{cr}$$

Considering the formulas (4) and (5), the critical level *d*_*cr*_ may be regarded as the quantile of the order *p*_cr_ of distribution describing the random variable *Z* = SPI and be written as ${d}_{cr}={u}_{{p}_{cr}}$. It is also possible to define the measure to calculate the value of critical level *d*_*cr*_ for the ‘a priori’ defined level of critical probability *p*_*cr*_*.* Since ${d}_{cr}={u}_{{p}_{cr}}$, then the quantile measure of risk is defined as:

**Definition 3.** The quantile measure of drought risk level defines the quantile of the order *p*_cr_ of the distribution of random variable *Z* = SPI, with a critical level to be exceeded with a flood risk at *p*_cr_ level, and is calculated using the formula:

8 $${D}_{Q}\left({G}_{{ }_{\gamma ,\mu ,\sigma }}^{-1},{p}_{cr}\right)={d}_{cr}={u}_{{p}_{cr}},$$

where ${G}_{{ }_{\gamma ,\mu ,\sigma }}^{-1}$ is the reverse distribution function of the generalized distribution of maximum values of random variable *Z* = SPI, *p*_cr_ is the probabilistic measure of drought risk described with the formula (6), and the ${u}_{{p}_{cr}}$ defined in the formula (5). Thus, an original probabilistic model of drought risk has been obtained which, in line with the authors’ intentions, is to be used for measuring and assessing the risk of drought-related damage. The model is defined by definition 4.

**Definition 4.** The probabilistic model of drought risk based on distribution function ${G}_{\gamma ,\mu ,\sigma }$ of the generalized distribution of maximums of random variable *Z* = SPI defining the drought level with the monthly SPI is defined by the formulas:

9 $${R}_{D}\left({G}_{\gamma ,\mu ,\sigma },{d}_{cr}\right)=D$$

and

10 $${R}_{DQ}\left({G}_{\gamma ,\mu ,\sigma }^{-1},{p}_{cr}\right)={D}_{Q}$$

*R*_*D*_ is the measure of the risk of the occurrence of random variable value *Z* = SPI below level *d*_cr_. *R*_*DQ*_ is the measure of the level below which the random variable value will reach *Z* = SPI with defined critical probability *p*_cr_.

## Results and discussion

**Fig. 1:**
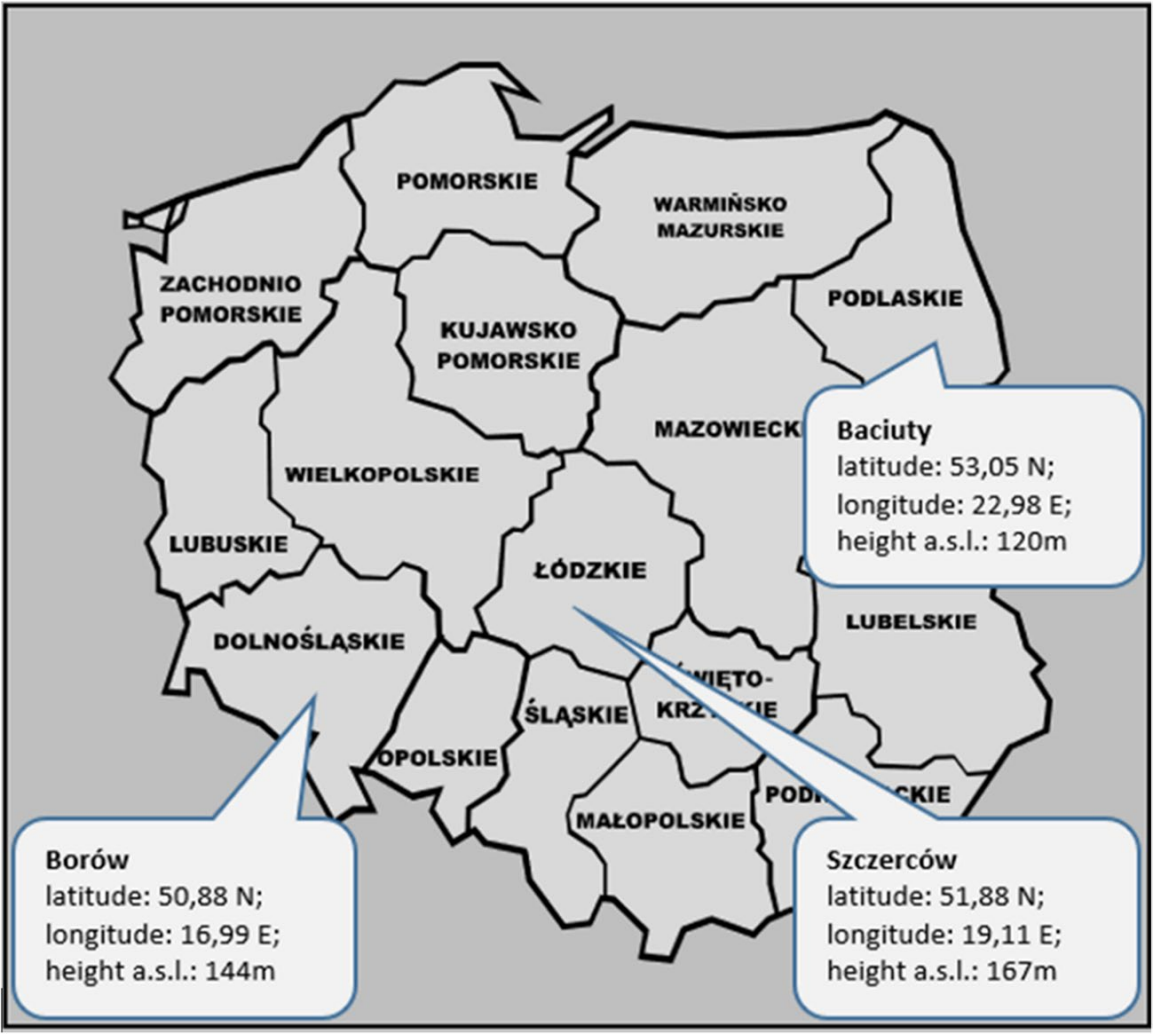
Location of measurement points

As mentioned in the “Introduction” section, the research was conducted in three different provinces in Poland: dolnośląskie, łódzkie, and podlaskie. The data was collected in three tier V precipitation stations located in Borów (dolnośląskie), Szczerców (łódzkie), and Baciuty (podlaskie) (Fig. 1).

The selection of these measurement points for studying drought in Poland was not accidental. The main criterion for the selection was the diversity in the duration of the growing season (average daily air temperature above 5 °C) in Poland. There is a line of decreasing growing season running from south-west Poland, through central Poland, all the way to the north-east. This means that dolnośląskie province, located in south-west Poland, has the longest growing season (about 235 days), while podlaskie province, located in north-east Poland, has the shortest growing season (about 205–210 days). Obviously, the growing season in łódzkie province (about 225 days) falls between the extreme values, since the province is located in central Poland (Tomczyk and Szyga-Pluta 2016). For this reason, the study was conducted using data collected from the three aforementioned provinces.

In each of the three locations, daily data from the period between 01 January 2000 and 31 December 2019 was collected and analysed. The time series for each location, taking into consideration planned drought risk analyses and dynamic climate change in the world in the last 20 years, were divided into four shorter 5-year series: period I (2000–2004), period II (2005–2009), period III (2010–2014), and period IV (2015–2019). The division of the analysed period into 5-year sub-periods and the selection of the maximum values from the monthly periods give samples with *n* = 60 maximum values. Such a sample size ensures the appropriate quality of the estimated parameters of the model. The division into 1-year periods with selection of monthly maxima would give us *n* = 12 samples, which is insufficient when using the stochastic approach. A monthly division is obviously impossible because the sample size would be *n* = 1. On the other hand, the period should not be increased, for example, to 15 years or 20 years, because there would be a problem of not taking into account the impact of climate change, which occurs over longer periods of time.

For each period in each of the studied locations, we estimated the parameters of theoretical distribution functions ${G}_{\gamma ,\mu ,\sigma }$ of the random variable *Z* = SPI as a standardized precipitation index, and correlated them with theoretical distribution functions on the same graphs. The estimation of stochastic drought risk models for 5-year periods allows the trend of changes in risk over the entire period under study to be captured. For each of the three locations studied, drought risk trends are presented based on the results of stochastic risk measurement using the proposed proprietary models.

Due to the fact that the subject literature indicates that the random variable $Y=\sqrt[3]{X}$ has an approximately normal distribution, and therefore the random variable* Z* has a normal standardized distribution, the authors of the study have additionally tried to match the normal distribution with the empirical distribution of the random variable* Z* for each period and location. The results of the estimation obtained for the distribution described with distribution function ${G}_{\gamma ,\mu ,\sigma }$ have been correlated with the results obtained for normal distribution.

To verify the matching of the theoretical and empirical distribution functions, compatibility tests were performed (the Anderson–Darling test and the Kolmogorov–Smirnov test). The* p* value of the performed Anderson–Darling tests was accepted as a measuring instrument for matching the theoretical distributions.

Using the probabilistic model of drought risk (9) for the set critical level *d*_*cr*_ of the standardized precipitation index (random variable* Z*), the probability of damage resulting from the random variable reaching values below the critical level *h*_*cr*_ was estimated. The level *d*_*cr*_ =  − 2 was accepted as critical in the study, which, according to the classification of drought level in Table 1, distinguishes very dry periods and extremely dry periods (SPI <  − 2).

The graphs of the empirical distribution function for the distribution of random variable* Z* were prepared according to the procedure proposed by Thomas and Reiss (2007).

Table 2 presents the results of estimation of the parameters for distribution functions ${G}_{\gamma ,\mu ,\sigma }$ of the distribution of random variable* Z*, in the studied locations for four periods (period I 2000–2004, period II 2005–2009, period III 2010–2014, and period IV 2015–2019). The last two columns of Table 2 present the *p* values for two compatibility tests (the Anderson–Darling test and the Kolmogorov–Smirnov test). The basis for the probabilistic model of drought risk proposed by the authors is the theoretical distribution function of the distribution of the standardized precipitation index. The* p* value was accepted as a measuring instrument due to the quality of its matching to the empirical distribution.

**Table 2:** Estimator values for parameters of distribution functions ${G}_{\gamma ,\mu ,\sigma }$ along with compatibility test results

City	Period	$\widehat{\gamma }$	$\widehat{\mu }$	$\widehat{\sigma }$	*P*_*v*_ _Anderson–Darling_	*P*_*v*_ _Kolmogorov-Smirnov_
Borów	I	− 0.136	− 0.409	0.921	0.863	0.848
	II	0.079	− 0.398	0.761	0.656	0.677
	III	− 0.372	− 0.31	1.062	0.339	0.319
	IV	− 0.222	− 0.379	0.969	0.998	0.997
Szczerców	I	− 0.128	− 0.413	0.914	0.987	0.972
	II	− 0.322	− 0.36	1.24	0.327	0.468
	III	− 0.242	− 0.334	1.285	0.377	0.369
	IV	− 0.135	− 0.612	0.923	0.98	0.898
Baciuty	I	− 0.255	− 0.366	0.983	0.961	0.906
	II	− 0.153	− 0.363	0.993	0.642	0.701
	III	− 0.188	− 0.181	0.993	0.923	0.756
	IV	− 0.225	− 0.188	0.852	0.972	0.955

Source: own study

When analysing the values of measuring instrument *p* value presented in Table 2 for distribution functions ${G}_{\gamma ,\mu ,\sigma }$, we can clearly see that—for each location and studied period—the estimated theoretical distribution is compatible with the empirical distribution of the standardized precipitation index at any significance level *α* < 0.327 (considering the results of the Anderson–Darling test), where such a low *p* value only appears several times. In most cases, very high values of *p* value (close to 1) prove that the theoretical distribution was matched very well (almost perfectly) by the authors with the empirical distribution of the standardized precipitation index. The obtained results successfully verify hypothesis H1.

Additionally, when comparing the values of *p* value for theoretical normal distributions matched to empirical distributions of standardized precipitation indexes (Table 3) with the values of *p* value for theoretical distributions with distribution function ${G}_{\gamma ,\mu ,\sigma }$ (Table 2), we can see that the generalized distributions of extreme values are better matched with empirical distributions of the standardized precipitation index than the normal distribution. It is worth noting that none of the normal distributions matched the empirical distributions of random variable* Z* are a precisely standardized normal distribution where estimators of the expected value and standard deviation are $\widehat{\mu }$ = 0 and $\widehat{\sigma }$ = 1 respectively, such as presented in the literature, e.g. Krishnamoorthy et al. (2008).

**Table 3:** Estimator values for parameters of distribution functions for normal distribution along with compatibility test results

City	Period	$\widehat{\mu }$	$\widehat{\sigma }$	*P*_*v*_ _Anderson–Darling_	*P*_*v*_ _Kolmogorov-Smirnov_
Borów	I	0.008	0.962	0.805	0.822
	II	0.018	0.946	0.628	0.597
	III	0.086	0.881	0.309	0.291
	IV	0.026	1.027	0.996	0.992
Szczerców	I	0.012	0.958	0.943	0.921
	II	0.057	0.893	0.287	0.613
	III	0.132	1.287	0.265	0.357
	IV	− 0.259	1.019	0.895	0.812
Baciuty	I	0.062	1.1	0.931	0.812
	II	0.061	1.059	0.505	0.533
	III	0.229	1.059	0.819	0.508
	IV	0.122	0.943	0.929	0.917

Source: own study

**Fig. 2:**
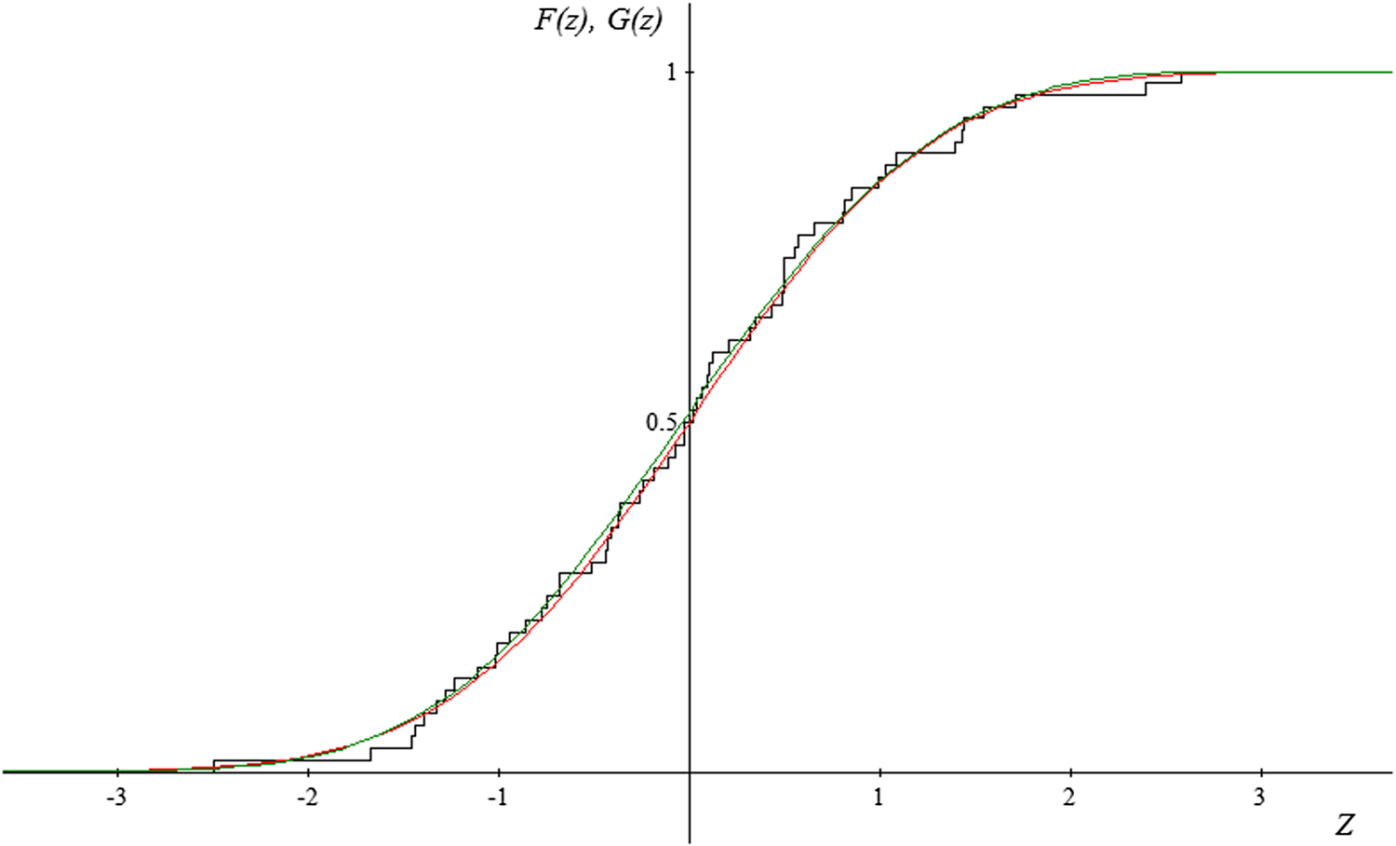
Graph of the empirical distribution function (black) for random variable* Z* for the period 2015—2019 in Borów, graphs of the matched distribution function of the distribution of extreme values *G*(*z*) (red), and graph of the matched distribution function of the normal distribution *F* (*z*) (green)

**Fig. 3:**
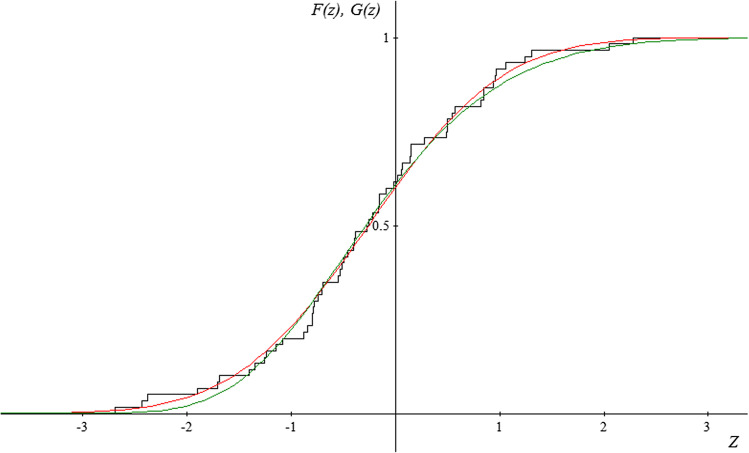
Graph of the empirical distribution function (black) for random variable* Z* for the period 2015–2019 in Szczerców, graphs of the matched distribution function of the distribution of extreme values *G*(*z*) (red) and graph of the matched distribution function of the normal distribution *F* (*z*) (green)

**Fig. 4:**
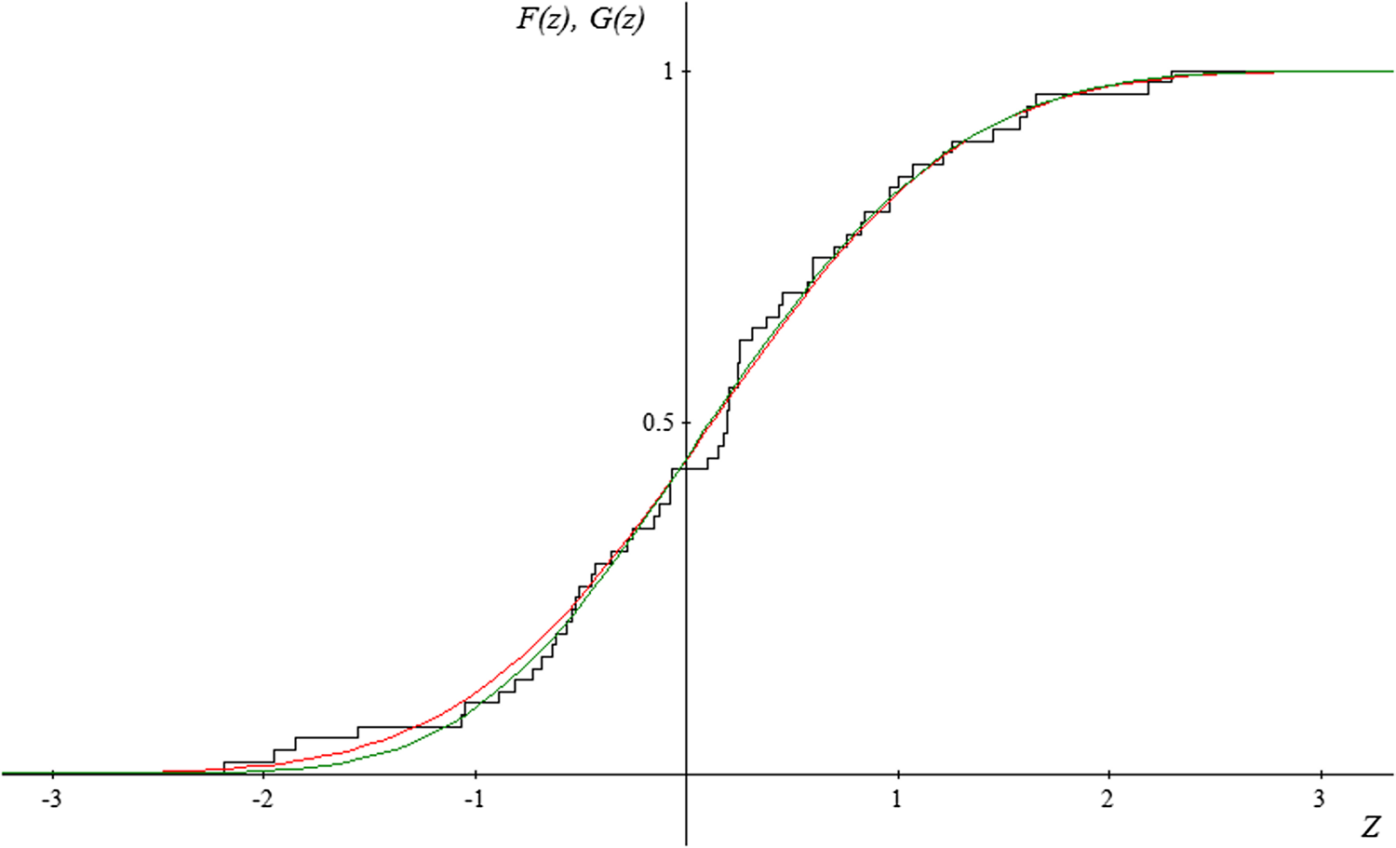
Graph of the empirical distribution function (black) for random variable* Z* for the period 2015–2019 in Baciuty, graphs of the matched distribution function of the distribution of extreme values *G*(*z*) (red) and graph of the matched distribution function of the normal distribution *F* (*z*) (green)

The figures presented below (Figs. 2, 3 and 4) show the course of the empirical distribution functions of the standardized precipitation index against the background of the theoretical distribution functions of the generalized extreme value distribution and normal distribution for the three locations in period IV (2015–2019).

**Fig. 5:**
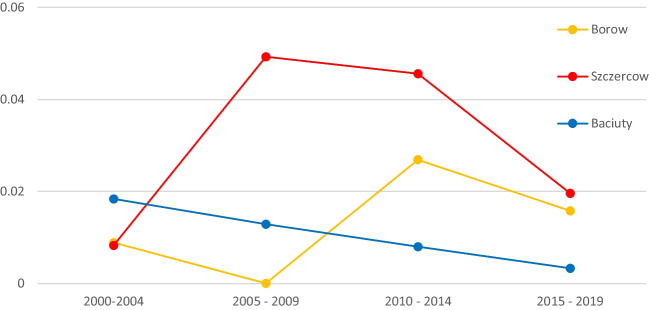
Graph of probabilistic measures of drought risk in the studied locations in the years 2000–2019

The results of the estimation of distribution parameters of the standardized precipitation index (Table 2) in the three studied provinces and the graphs of estimated probabilistic measures of drought risk (Fig. 5) indicate that, for each of the studied locations in the analysed period, different trends formed, both in terms of climatic trends and the nature of drought.

Analysis of the estimated parameters of distribution functions for the distribution of random variable* Z*, reflecting the value of the standardized precipitation index, showed clear differences in parameter values, both in terms of the location and the studied periods. The expected value of the generalized distribution of maximums is functionally dependent on location parameter *µ*, variability parameter *σ* and shape parameter *γ*. Therefore, this allows some conclusions to be drawn from the estimation of the theoretical distribution functions.

Over the last 20 years in Borów (dolnośląskie province), the estimator of location parameter *µ* has slightly increased in value, as has the estimator of variability parameter *σ*. In the case of the location parameter, this means that throughout the entire period of the study, the average value of the standardized precipitation index for this region has slightly increased, as has the uncertainty about its value, which is indicated by the increase in the value of the scale parameter. In the case of the second studied location (Szczerców, łódzkie province), a significant increase has been observed in the value of the estimator for parameter *µ* over the course of 20 years, with a simultaneous slight increase in the value of the estimator for scale parameter *σ*. The estimator values obtained for this location indicate a clear increase in the value of the standardized precipitation index and a slight increase in uncertainty about its value. It is worth noting that for periods II and III in this location, the values of the estimators of parameter *µ* were lower than for periods I and IV, whereas the values of the estimators of parameter *σ* were clearly higher than for periods I and IV. This means that in middle periods (II and III), the average values of the standardized precipitation index were lower, but were characterized by higher uncertainty about their value in comparison to the boundary periods analysed (I and IV). The results obtained from the third location (Baciuty) indicate that over the course of 20 years, a clear growth trend has been observed in the values of the estimators of parameter *µ*, with the value of the estimators of scale parameter *σ* in the last period (IV) reaching its lowest value (this successfully verifies hypothesis H2).

In terms of drought risk analysis, a significant parameter is the gamma parameter. Statistically, its value describes the properties of tails in the distribution of the initial variable—in this study random variable *Z* = SPI. The above values of parameter *γ* denote thick tails of distributions of the reference variables. This means that the higher the positive value of parameter *γ*, the thicker the right tail of distribution and, consequently, the increase in the probability of extreme phenomena. A zero value of shape parameter *γ* means a thin tail of distribution. Values below zero for parameter *γ* mean cut distributions where some extreme values cannot be reached by the random variable. Additionally, it should be noted that the lower the value of parameter *γ* below zero, the more the thickness of the left tail of distribution increases, along with the probability of extreme phenomena for the left side of the distribution. In other words, the probability of the studied variable reaching extreme minimum values increases.

Analysing the values of the estimators of parameter *γ* in each of the three study locations and each period, it was observed that the parameter has negative values in all but the second period in Borów. The results indicate that the estimated distributions of the standardized precipitation index in the studied locations and periods are cut distributions. This means that the studied standardized precipitation index may not reach certain maximum values. The obtained results confirm the actual rainfall phenomena. It is difficult to imagine rainfall with unlimited levels.

The analyses of the values of the extreme index (parameter *γ*) in the three studied regions over the course of 20 years have generated an image for each region:

- Borów: the value of parameter *γ* in the last period has a lower value than in the first period; therefore, it can be stated that the probability of extreme drought phenomena has increased over the course of the analysed period. The lowest value of parameter *γ* was recorded in period III, which means that the probability of extreme drought was highest in that period.
- Szczerców: in this location, the value of the estimator of parameter γ in periods I and IV is clearly higher than the value in periods II and III. This means that the probability of extreme drought is significantly higher in the middle periods than in the boundary periods of the entire duration of the study. Additionally, the probability of extreme drought is higher in period IV than in period I.
- Baciuty: in this location, we observed a decreasing trend in the values of the estimators of the extreme index over periods II to IV. This indicates an increasing trend in the probability of extreme drought. The value of the estimator of the extreme index is higher in period I than any other period; therefore in period I, the probability of extreme drought was the highest.

In addition to the aforementioned analyses of the values of the estimators of parameter *γ*, it is essential to note that the analyses were conducted in *ceteris paribus* conditions. This means that the inference is only true with unchanged values of the other parameters of the distribution of the standardized precipitation index, responsible for the probability of extreme drought.

The original probabilistic model of measuring the risk of drought-related socio-economic damage was used to calculate the drought risk level for the three regions in the studied periods. The calculations of drought risk levels are presented in Table 4. The colour scale of the risk levels was developed on the basis of the flood risk level scale developed by Kuźmiński (2018).

**Table 4:**
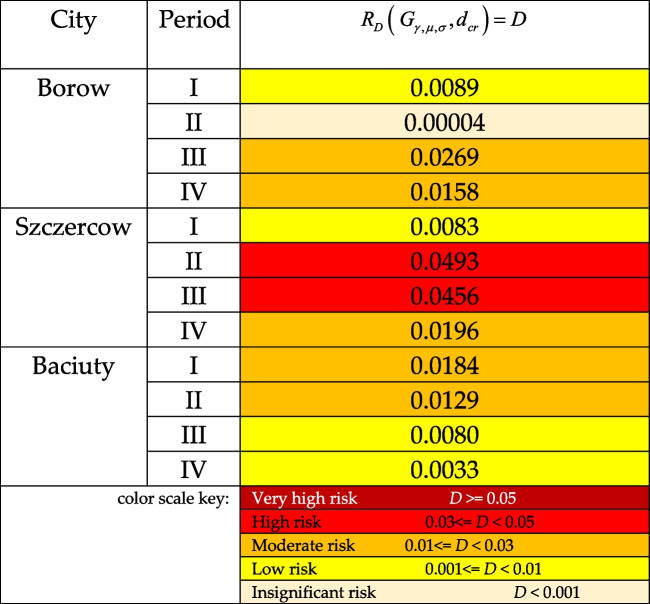
Measures of drought risk for individual regions in periods I–IV, estimated with the probabilistic model of drought risk

Initial analysis of the results of probabilistic measures of drought risk for the studied regions in the studied periods indicates that each region is characterized by completely different drought risk characteristics over the 20-year period (2000–2019).

Analysis of the results of drought risk estimates for the study periods showed that period I witnessed the highest risk in podlaskie province, while łódzkie and dolnośląskie provinces recorded similar drought risk levels. Period II had the lowest drought risk in dolnośląskie province, then podlaskie province, with the highest drought-related threat risk recorded in łódzkie province. In period III, again, łódzkie province came first in terms of drought risk level, then dolnośląskie province, and podlaskie last. In period IV, the region with the highest drought risk was łódzkie province, dolnośląskie province came second, and the safest in terms of drought risk was podlaskie province.

When analysing the studied regions in terms of drought risk levels, it is worth noting that the safest region is podlaskie province, for which a clear linear declining trend is observed over the studied period of 20 years in terms of drought risk (Fig. 5).

In case of łódzkie province, a clear increase in drought risk was observed in periods II and III compared to periods I and IV. Over the course of 20 years, łódzkie province has recorded an increase in drought risk level.

When it comes to dolnośląskie province, a clear decrease in drought risk level was observed in period II compared to period I, and, simultaneously, a clear increase in the risk level in periods III and IV compared to period II.

The study results presented in this section provide grounds for successful verification of hypotheses H3 and H4.

## Conclusion

Three factors can be distinguished which determine drought risk and drought-related damage risk levels: frequency, scale and variability of events. A combination of the three factors generates specific values for the probability of extreme phenomena. The values are regarded as probabilistic measures of drought-related damage risk. The probability, meaning the probabilistic measure of drought risk, being the combined result of three parameters of theoretical distribution, is the main component of the probabilistic model of drought risk described in this paper. Moreover, the stochastic approach to drought-related damage risk assessment helps to characterize the studied regions over the studied periods in terms of the threat.

The proprietary models presented in this study for the probabilistic quantification of extreme drought-related damage risk are universal in nature. This means that they can be used to assess the risk of the phenomena in regions located in different climatic zones. The presented application of the probabilistic approach to the problem of drought risk assessment may constitute a basis for the development of indexes for any given climatic region (balance catchment areas, water regions, etc.). The universal nature of the models results from the fact they are flexible due to three replaceable and regulated components: a modelled variable describing a phenomenon, the critical level of the modelled variable and the theoretical distribution function of the modelled variable. In this study, the authors have presented a model where the modelled variable is the standardized precipitation index (SPI), the critical level was the value of the index above which extreme drought is observed and the distribution function of the generalized extreme value distribution was accepted as the theoretical distribution function describing the possible level of SPI. Each of the components is replaceable, which makes the entire model highly flexible and suitable for a wide variety of applications. The developed models fill a methodological gap in the field of measuring and assessing the risk of natural phenomena of an extreme nature. The models can be used by a broad group of scientists and practitioners, filling a gap in research applications.

The created model may form a significant element of an active environmental and economic policy regarding the occurrence of drought, and can help in identification and typification of regions exposed to the effects of drought. The calculations may form a basis for making strategic, tactical and operational decisions related to water management: (1) forecasting the intensity of drought in farming areas; (2) creating environmental scenarios related to climate change; and (3) developing structures for drought risk management, etc.

It is also worth pointing out the limitations of using the presented models. As indicated above, one of the fundamental components of the models is the distribution function of the distribution of the modelled variable. A number of different random variables may be used to describe extreme natural phenomena. These are usually meteorological characteristics (e.g. temperature, precipitation, water flow) or hydrological characteristics (water level, water flow). The basic requirement for using probabilistic models of risk measurement is to find the theoretical distribution function of the distribution of the variable (characteristic) describing the studied phenomenon, which is matched to the empirical distribution of the said characteristic at the appropriate level. There may be difficulties in finding the theoretical distribution and the estimation of its parameters. In response to the problems described above, the creators of the models have made it possible to use the distribution function of any distribution, and any method of estimating its parameters.

The rationality behind the proposed method is based mainly on the very high quality of the proposed models. Assessment of the quality of the proposed drought risk models was carried out using assessment of the fit of the theoretical distribution in the drought risk model to the empirical distribution of the values of maximum daily precipitation. As a measure of fit, the authors adopted the *p* value of the test of consistency between the theoretical distributions and empirical distributions of the characteristics under study. Both tests (Anderson–Darling and Kolmogorov–Smirnov) gave very good results (high *p* values), which proves the high quality of the results obtained from the models of probabilistic quantification of the drought risk level.

## Data Availability

The datasets used and analysed during the current study are available from the corresponding author on reasonable request.

## References

[CR1] Arns A, Wahl T, Haigh I, Jensena J, Pattiaratchi C (2015). Estimating extreme water level probabilities: a comparison of the direct methods and recommendations for best practice. Coast Eng.

[CR2] Ayantobo OO, Li Y, Song S, Javed T, Yao N (2018). Probabilistic modelling of drought events in China via 2-dimensional joint copula. J Hydrol.

[CR3] Beguería S, Vicente-Serrano SM, Reig F, Latorre B (2014). Standardized precipitation evapotranspiration index (SPEI) revisited: parameter fitting, evapotranspiration models, tools, datasets and drought monitoring. Int J Climatol.

[CR4] Blauhut V, Stahl K, Stagge JH, Tallaksen LM, De Stefano L, Vogt J (2016). Estimating drought risk across Europe from reported drought impacts, drought indices, and vulnerability factors. Hydrol Earth Syst Sci.

[CR5] Boergens E, Güntner A, Dobslaw H, Dahle C (2020). Quantifying the Central European droughts in 2018 and 2019 with GRACE Follow-On. Geophys Res Lett.

[CR6] Brito SSB, Cunha APM, Cunningham CC, Alvalá RC, Marengo JA, Carvalho MA (2018). Frequency, duration and severity of drought in the Semiarid Northeast Brazil region. Int J Climatol.

[CR7] Burke EJ, Perry RH, Brown SJ (2010). An extreme value analysis of UK drought and projections of change in the future. J Hydrol.

[CR8] Charon C (2015). Probability distributions of wind speed in the UAE. Energy Convers Manag.

[CR9] Costa AC (2011). Local patterns and trends of the standard precipitation index in southern Portugal (1940–1999). Adv Geosci.

[CR10] Dai M, Huang S, Huang Q, Leng G, Guo Y, Wang L, Fang W, Li P, Zheng X (2020). Assessing agricultural drought risk and its dynamic evolution characteristics. Agric Water Manag.

[CR11] Engeland K, Frigessi A, Hisdal H (2005). Practical extreme value modelling of hydrological floods and droughts: a case study. Extremes.

[CR12] Felbermayr G, Gröschl J, Sanders M, Schippers V, Steinwachs T (2022). The economic impact of weather anomalies. World Dev.

[CR13] Findlay A (2020). Six centuries of drought. Nat Clim Chang.

[CR14] Fisher R, Tippett L (1928). Limiting forms of the frequency distribution of the largest or smallest members of a sample. Proc Camb Philos Soc.

[CR15] Garreaud RD, Boisier JP, Rondanelli R, Montecinos A, Sepúlveda HH, Veloso-Aguila D (2020). The central Chile mega drought (2010–2018): a climate dynamics perspective. Int J Climatol.

[CR16] Geng SM, Yan DH, Zhang TX, Weng BS, Zhang ZB, Qin TL (2015). Effects of drought stress on agriculture soil. Nat Hazards.

[CR17] Gepert E (1971) Problem ryzyk wielkich i katastrofalnych. *Wiadomości Ubezpieczeniowe* 1 [In Polish]

[CR18] Gomes M, Guillou A (2015). Extreme value theory and statistics of univariate extremes: a review. Int Stat Rev.

[CR19] Guo Y, Huang S, Huang Q, Leng G, Fang W, Wang L, Wang H (2020). Propagation thresholds of meteorological drought for triggering hydrological drought at various levels. Sci Total Environ.

[CR20] Gupta A, Rico-Medina A, Caño-Delgado AI (2020). The physiology of plant responses to drought. Science.

[CR21] Hao Z, Hao F, Singh VP, Ouyang W, Cheng H (2017). An integrated package for drought monitoring, prediction and analysis to aid drought modeling and assessment. Environ Model Softw.

[CR22] Herman J, Quinn J, Steinschneider S, Giuliani M, Fletcher S (2020). Climate adaptation as a control problem: review and perspectives on dynamic water resources planning under uncertainty. Water Resour Res.

[CR23] Hosking JRM, Wallis JR, Wood EF (1985). Estimation of the generalized extremevalue distribution by the method of probability-weighted moments. Technometrics.

[CR24] Johnson N, Kotz S (1970) Distributions in Statistics. Continous Univariate Distributions, vol. 1, 2^nd^ edition. Boston: Houghton Mifflin

[CR25] Keyantash J, Dracup JA (2002). The quantification of drought: an evaluation of drought indices. Bull Am Meteor Soc.

[CR26] King AD, Pitman AJ, Henley BJ, Ukkola AM, Brown JR (2020). The role of climate variability in Australian drought. Nat Clim Chang.

[CR27] Kotz S, Nadarajah S (2005). Extreme value distributions. Theory and Applications.

[CR28] Krishnamoorthy K, Mathew T, Mukherjee S (2008). Normal-based methods for a gamma distribution: prediction and tolerance intervals and stress-strenght reliability. Technometrics.

[CR29] Kuźmiński Ł (2018) Modele probabilistycznego pomiaru i oceny ryzyka powodziowego na przykładzie dorzecza środkowej Odry. Wrocław: Wydawnictwo Uniwersytetu Ekonomicznego we Wrocławiu [In Polish]

[CR30] Łabędzki L (2006) Susze rolnicze. Zarys problematyki oraz metody monitorowania i klasyfikacji. Woda Środowisko Obszary Wiejskie. Rozprawy Naukowe i Monografie, pp. 107 [In Polish]

[CR31] Langat PK, Kumar L, Koech R (2019). Identification of the most suitable probability distribution models for maximum, minimum, and mean streamflow. Water.

[CR32] Lin Y, Deng X, Jin Q (2013). Economic effects of drought on agriculture in North China. Int J Disaster Risk Sci.

[CR33] Maity R, Suman M, Verma NK (2016). Drought prediction using a wavelet based approach to model the temporal consequences of different types of droughts. J Hydrol.

[CR34] Matthys G, Beirlant J (2001) Extreme quantile estimation for heavy-tailed distributions. Working paper, University Center of Statistics, Katholieke University Leuven. http://www.kuleuven.ac.be/ucs/research/publi.htm

[CR35] McKee TB, Doesken N, Kleist J (1993) The relationship of drought frequency and duration to time scale. In: Proceedings of the eighth conference on applied climatology, Anaheim, California, 17–22 January 1993. Boston, American Meteorological Society, pp 179–184. https://climate.colostate.edu/pdfs/relationshipofdroughtfrequency.pdf

[CR36] McNeil A (1998) Calculating quantile risk measures for financial time series using extreme value theory. Zurich: ETH. 10.3929/ethz-a-004320029

[CR37] Mishra AK, Singh VP (2010). A review of drought concepts. J Hydrol.

[CR38] Mishra AK, Singh VP, Desai VR (2009). Drought characterization: a probabilistic approach. Stoch Env Res Risk Assess.

[CR39] Oh H, Kim HJ, Mehboob MS, Kim J, Kim Y (2023). Sources and uncertainties of future global drought risk with ISIMIP2b climate scenarios and socioeconomic indicators. Sci Total Environ.

[CR40] Paulo AA, Pereira LS (2006). Drought concepts and characterization: comparing drought indices applied at local and regional scales. Water Int.

[CR41] Powers JS, Vargas GG, Brodribb TJ, Schwartz NB, Pérez-Aviles D, Smith-Martin CM, Becknell JM, Aureli F, Blanco R, Calderón-Morales E, Calvo-Alvarado JC, Calvo-Obando AJ, Chavarría MM, Carvajal-Vanegas D, Jiménez-Rodríguez CD, Chacon EM, Schaffner CM, Werden LK, Xu X, Medvigy D (2020). A catastrophic tropical drought kills hydraulically vulnerable tree species. Glob Change Biol.

[CR42] Schuldt B, Buras A, Arend M, Vitasse Y, Beierkuhnlein C, Damm A, Gharun M, Grams TEE, Hauck M, Hajek P, Hartmann H, Hiltbrunner E, Hoch G, Holloway-Phillips M, Körner C, Larysch E, Lübbe T, Nelson DB, Rammig A, Rigling A, Rose L, Ruehr NR, Schumann K, Weiser F, Werner C, Wohlgemuth T, Zang CS, Kahmen A (2020). A first assessment of the impact of the extreme 2018 summer drought on Central European forests. Basic Appl Ecol.

[CR43] Soule PT (1990) Spatial patterns of multiple drought types in the contiguous United States: a seasonal comparison. Clim Res 1:13–21

[CR44] Spinoni J, Vogt JV, Naumann G, Barbosa P, Dosio A (2018). Will drought events become more frequent and severe in Europe?. Int J Climatol.

[CR45] Thomas M, Reiss R (2007). Statistical Analysis of Extreme Value with Applications to Insurance, Finance, Hydrology and Other Fields.

[CR46] Tigkas D, Vangelis H, Tsakiris G (2017). An enhanced effective reconnaissance drought index for the characterisation of agricultural drought. Environ Processes.

[CR47] Tigkas D, Vangelis H, Tsakiris G (2019). Drought characterisation based on an agriculture-oriented standardised precipitation index. Theoret Appl Climatol.

[CR48] Tomczyk AM, Szyga-Pluta K (2016). Growing seasons in Poland in the period 1971–2010. Przegląd Geograficzny.

[CR49] Tsakiris G, Pangalou D, Vangelis H (2007). Regional drought assessment based on the Reconnaissance Drought Index (RDI). Water Resour Manag.

[CR50] Vicente-Serrano SM, Beguería S, López-Moreno JI (2010). A multiscalar drought index sensitive to global warming: the standardized precipitation evapotranspiration index. J Clim.

[CR51] Von Mises R (1936). La distribution de la plus grande de n valeurs. Rev Math Union Interbalcaniqu.

[CR52] Williams A, de Vries FT (2020). Plant root exudation under drought: implications for ecosystem functioning. New Phytologis.

[CR53] Williges K, Mechler R, Bowyer P, Balkovic J (2017). Towards an assessment of adaptive capacity of the European agricultural sector to droughts. Clim Serv.

[CR54] Wu J, Miao C, Zheng H, Duan Q, Lei X, Li H (2018). Meteorological and hydrological drought on the Loess Plateau, China: evolutionary characteristics, impact, and propagation. J Geophys Res: Atmospheres.

[CR55] Xu Z, Wu Z, Guo X, He H (2023). Estimation of water required to recover from agricultural drought: Perspective from regression and probabilistic analysis methods. J Hydrol.

[CR56] Xu L, Wang H, Chen J (2011) Application of extreme value analysis to extreme drought disaster area in China. In Modeling Risk Management for Resources and Environment in China. Springer Berlin Heidelberg, pp. 349–357. 10.1007/978-3-642-18387-4_39

[CR57] Yang W, Zhang L, Gao Y (2023). Drought and flood risk assessment for rainfed agriculture based on Copula-Bayesian conditional probabilities. Ecol Indic.

[CR58] Zarch MAA, Sivakumar B, Sharma A (2015). Droughts in a warming climate: a global assessment of Standardized precipitation index (SPI) and Reconnaissance drought index (RDI). J Hydrol.

[CR59] Zargar A, Sadiq R, Naser B, Khan FI (2011). A review of drought indices. Environ Rev.

[CR60] Ziolkowska JR (2016). Socio-economic implications of drought in the agricultural sector and the state economy. Economies.

